# IgG4‐related inflammatory pancreatic head pseudotumor mirrors pancreatic head tumor: A novel case series with a review of the literature

**DOI:** 10.1002/ccr3.8467

**Published:** 2024-02-04

**Authors:** Faheemullah Khan, Jehanzeb Shahid, Amna Saleem, Uzzam Ahmed Khawaja, Wasim Ahmed Memon, Uffan Zafar, Tariq Abdul Hameed, Khabab Abbasher Hussien Mohamed Ahmed

**Affiliations:** ^1^ Department of Radiology Aga Khan University Hospital Karachi Pakistan; ^2^ Jinnah Medical and Dental College Karachi Pakistan; ^3^ Department of Radiology Indiana University School of Medicine Indianapolis Indiana USA; ^4^ Faculty of Medicine University of Khartoum Khartoum Sudan

**Keywords:** abdominal CT, pancreatic tumor, pseud tumor, serum IgG4

## Abstract

**Key Clinical Message:**

In this noteworthy case series regarding pancreatic pseudo tumors, we intend to spread knowledge among physicians for the diagnostic and therapeutic approach and eventual disease prognosis.

**Abstract:**

Inflammatory pseudotumor of pancreatic head greatly mimics pancreatic head tumor. One of them is IgG4‐related pancreatic disease, which is commonly mistaken as neoplastic disease on imaging. In our novel case series, we report three cases of IgG4‐related pancreatic head pseudotumor with patients ranging from 35 to 72 years of age. Patients presented with jaundice and abdominal pain. Alongside initial laboratory workup, abdominal CTs and serum IgG4 levels were also obtained. Imaging features in conjunction with IgG4 levels confirmed the diagnosis of IgG4‐related autoimmune pancreatitis. Pancreatic pseudotumors are notorious for being often reported as real tumors. Through our noteworthy case series, we intend to highlight the imaging features and laboratory markers that are crucial in such cases to avoid invasive procedures.

## 1 INTRODUCTION

Radiologists are finding it difficult and challenging to differentiate between the different pancreatic pathologies as the diverse range of diseases mimics pancreatic neoplasm.^1^, ^2^, ^3^ Such pathologies are commonly named pancreatic pseudotumors. Multiple illnesses fall under the umbrella of the pancreatic pseudo‐tumors including autoimmune pancreatitis, intra‐pancreatic accessory spleen, abrupt pancreatic hemorrhage, chronic pancreatitis, groove pancreatitis, and fatty replacement of the pancreas.^4^, ^5^, ^6^ Non‐cancerous lesions, often indicated as pseudo‐tumors are revealed in around 5%–10% of patients undergoing pancreatectomies on account of imaging appearance of these entities being mistaken for pancreatic tumor.^7^, ^8^

Inflammatory pseudotumor (IPT) has been described in the literature as a non‐neoplastic space‐occupying lesion that may occur almost anywhere in the body (including the pancreas) and has a varying course with various clinical presentations as well as multiple radiological and histological patterns. The usual pathogenesis of this disease is secondary to chronic inflammatory conditions encompassing genetic or developmental defects, infections, physical injuries, and lymphoproliferative disorders.^8^, ^9^ IgG4‐related disease also falls in the category of IPT.

Pancreatic resection in these cases is mainly due to the preoperative diagnostic difficulty that is resolved surely only with histopathological examination of the specimen.^10^ Given the subacute presentation of IgG4‐related disease, laboratory and radiological investigations are of paramount importance in reaching the correct diagnosis.^10^

Herein, we present a set of patients presenting with a non‐neoplastic mass in the pancreas on abdominal CT scan indicative of a pancreatic pseudotumor, in current case series IgG4 related disease. The purpose is to highlight the imaging features and laboratory markers of autoimmune pancreatitis/IgG4‐related pancreatic disease that help to differentiate this entity from a true pancreatic tumor.

## 2 CASE PRESENTATION

### 2.1 Case 1

**FIGURE 1:**
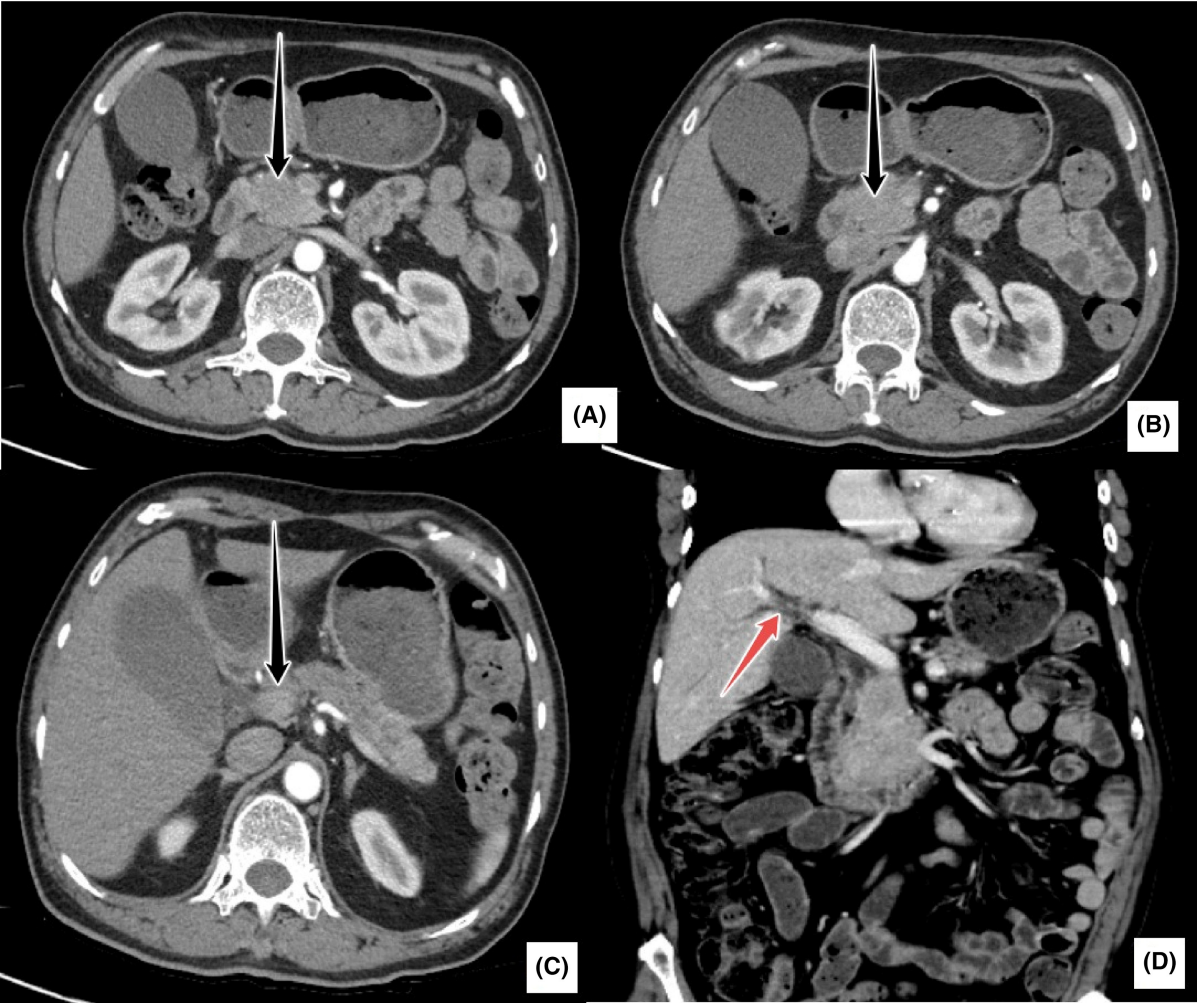
72‐year‐old male with the indication of pancreatic mass. Serum IgG4 levels were elevated at 2220 mg/L. (A–C) Images from abdominal contrast‐enhanced CT (CECT) showing a large mildly enhancing, lobulated, soft tissue mass involving the head and uncinate process of the pancreas (black arrows). (D) Note that despite a large pancreatic head mass, there is only minimal intrahepatic biliary dilatation (red arrow).

A 72‐year‐old male patient with comorbidities including diabetes mellitus, ischemic heart disease and benign prostatic hyperplasia came to our hospital with complaints of yellow discolouration of sclera, dark‐colored urine and pale‐colored stools for 15 days. Patient history included coronary artery bypass grafting (18 years ago) and carotid endarterectomy (19 years ago). On arrival, the patient was stable except for having jaundice. CT scan abdomen with contrast showed a heterogeneous appearing lesion involving the uncinate process of the pancreas reported as a neoplastic lesion (Figure 1). A few small pulmonary pleural‐based soft tissue density nodules were also noted as suspicious for metastatic deposits. His blood CA19‐9 was 42.07 U/mL (Normal: non‐detectable to 39 U/mL). PET‐CT showed low FDG avid soft tissue lesions involving the uncinate process of the pancreas and non‐FDG avid predominantly subpleural subcentimeter bilateral lung nodules. The patient underwent a Whipple procedure (pancreaticoduodenectomy) under general anesthesia. Histopathology reported no evidence of malignancy in the surgical specimen.

Post‐procedure, the patient was shifted to the intensive care unit for postsurgical care. He became hypotensive and therefore was kept on dual inotropic support. The patient had a raised troponin level of 25 and developed atrial fibrillation. He was managed with intravenous fluids, analgesics, antibiotics, Vitamin K, Amiodarone and anti‐emetics. Chest radiograph revealed left‐sided pleural effusion which was aspirated under ultrasound guidance. His Jackson Pratt drain was removed on the 9th postoperative day. The patient had complaints of bloating and abdominal pain, therefore CT scan abdomen and pelvis with contrast was done, which did not report any anastamotic leak or abdominal collection. His Jackson Pratt drain was then removed on the 9th postoperative day. He was eventually mobilized out of bed. Incentive spirometry and chest physiotherapy were done. His diet progressed gradually which he tolerated well. The patient was in stable condition at the time of discharge. Serum IgG4 levels performed 2 days before discharge were elevated at 2220 mg/L (39.2–864). Based on the negative histopathology report for malignancy and raised serum IgG4 levels, the final diagnosis was that of IgG4‐related autoimmune pancreatitis. The patient was put on steroids. The patient's follow‐up CT scan abdomen after 1 year was unremarkable. Serum IgG4 levels were 1470 mg/L. The patient had no complaints of pain or jaundice.

### 2.2 Case 2

**FIGURE 2:**
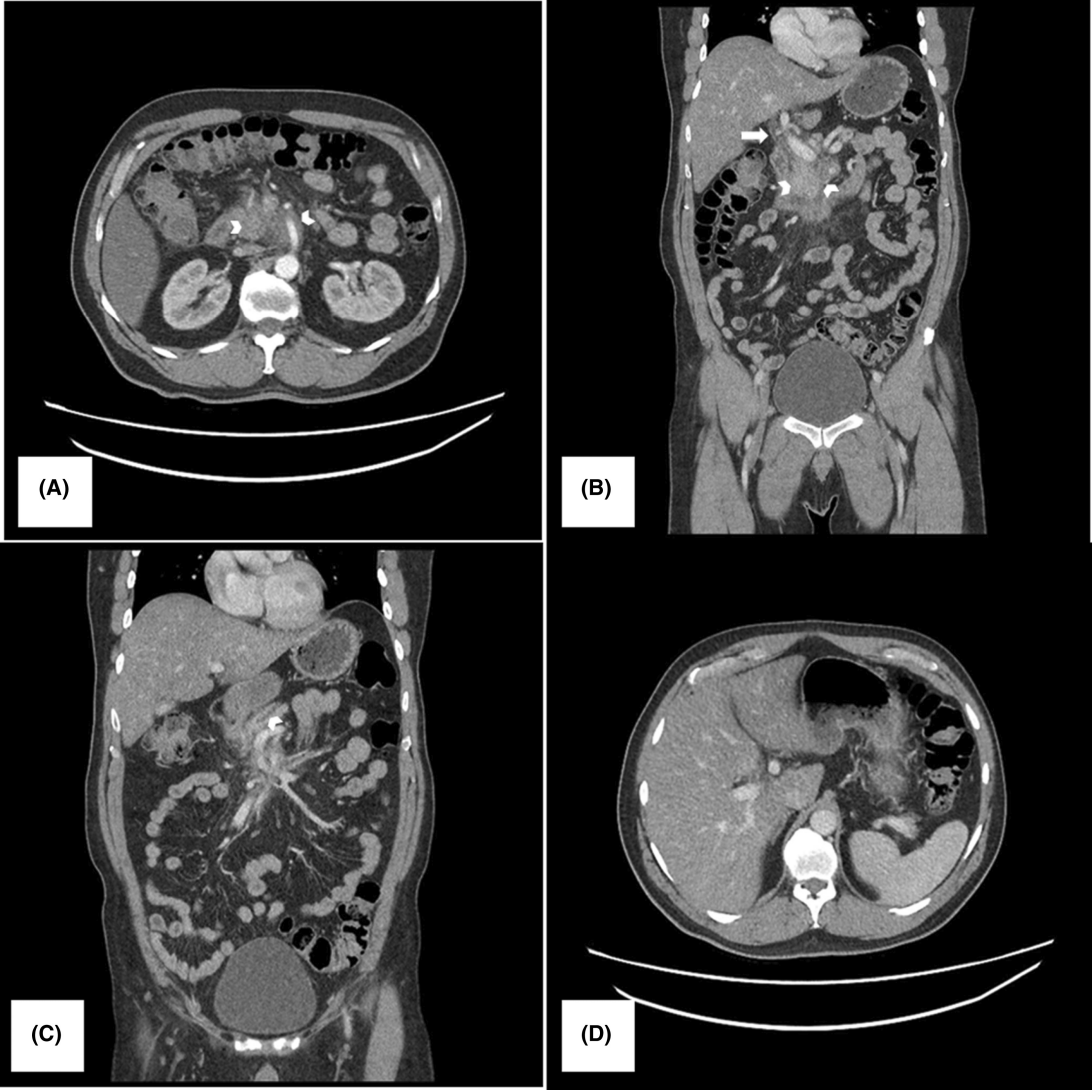
52‐year‐old male whose CT abdomen examination was requested with the indication “hepatic steatorrhea and left parapelvic cyst”. Serum IgG4 levels elevated to 1130 mg/L. (A) Abdominal contrast‐enhanced CT abdomen (CECT) axial section, showing a mildly enhancing soft tissue density mass involving the uncinate process of pancreas and also completely encasing superior mesenteric artery (white arrowhead). (B) Coronal section from CECT. Showing that mass is also encasing the pancreatic head, uncinate process and distal common bile duct (CBD) (white arrowhead). In addition to, there is the faint enhancement of the walls of CDB as well, one of the ancillary findings in IgG‐4‐related disease (white arrow). (C) Another coronal section of CECT at the level of the pancreatic duct. There is borderline dilatation of the pancreatic duct (PD) measuring 2.8 mm (white arrowhead). However, there is no intrahepatic biliary dilatation. (D) Axial section from CECT, showing no intrahepatic biliary dilation despite distal mass.

A 52‐year‐old male patient, with a known case of hypertension and diabetes presented to our radiology department as an outside referral with the indication of “hepatic steatorrhea and left parapelvic cyst”. The referral was for a CT abdomen with contrast which showed mildly enhancing soft tissue density mass involving the head and uncinate process of the pancreas and completely encasing the superior mesenteric artery. No associated intra or extra‐hepatic biliary dilatation and no peripancreatic lymphadenopathy (Figure 2). Serum IgG4 levels were raised (1130 mg/L) supporting IgG4‐related pancreatic disease. The patient was put on steroids. The patient did not show up for follow‐up imaging as he was asymptomatic.

### 2.3 Case 3

**FIGURE 3:**
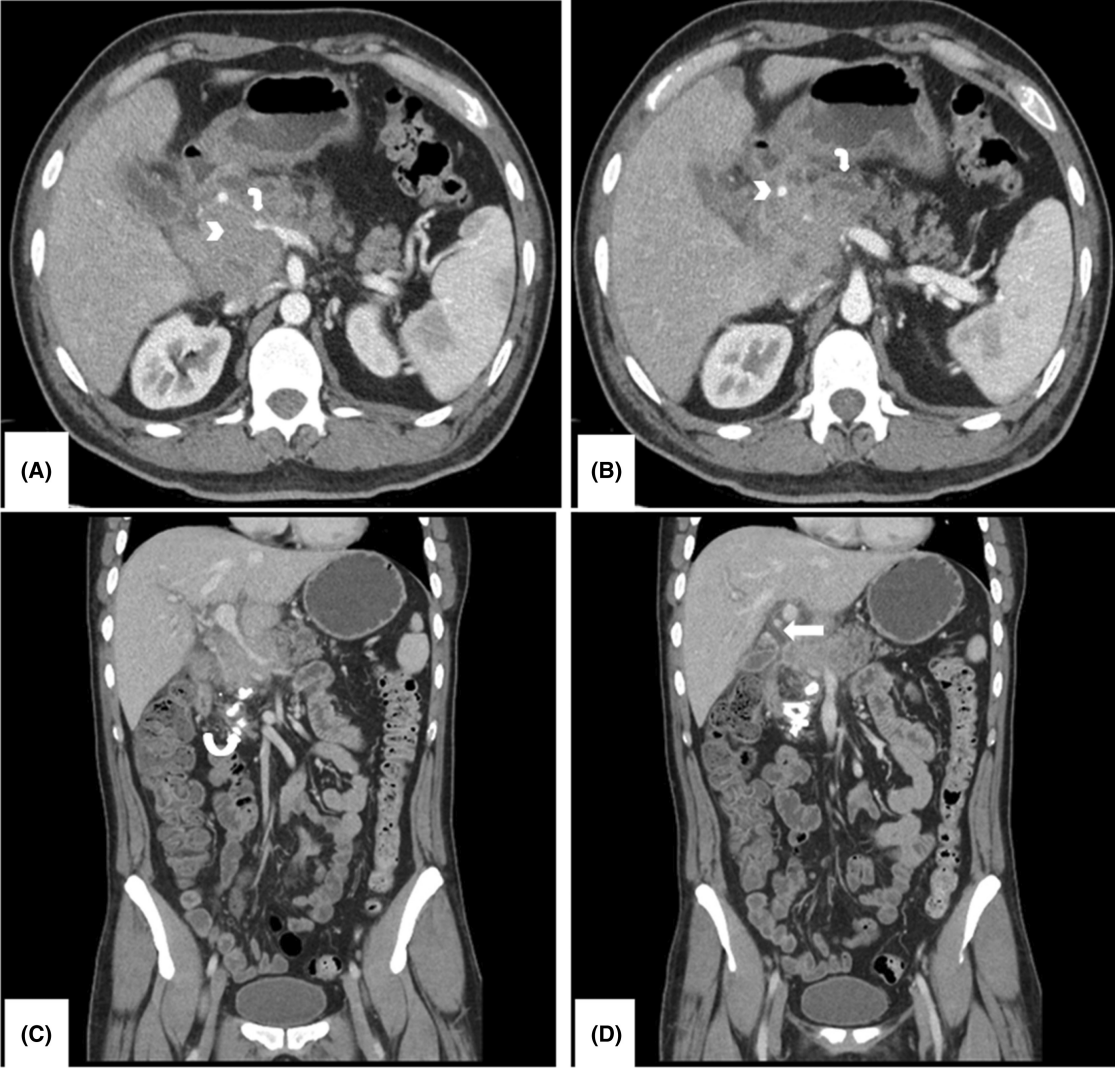
A 39‐year‐old male CT abdomen examination was requested with a history of recurrent pancreatitis. Serum IgG4 levels were elevated to 2960 mg/L. (A) Axial section from abdominal CECT showing a mildly enhancing but heterogeneous, soft tissue density mass involving the head of the pancreas (white arrowhead), which is completely encasing the portal vein resulting in its narrowing (bent arrow). (B) Axial section from CECT showing that mass is encasing the hepatic artery properly (white arrowhead). There is mild dilatation of the pancreatic duct measuring at 3.5 mm (bent arrow). (C) Coronal section from CECT again showing significant portal vein narrowing without thrombosis. Foci of coarse calcification is seen in the head and uncinate process, in keeping with chronic pancreatitis (circular arrow). (D) Coronal section from CECT showing faint enhancement of the common hepatic duct, one of the ancillary findings seen with IgG4‐related disease (white arrow).

**FIGURE 4:**
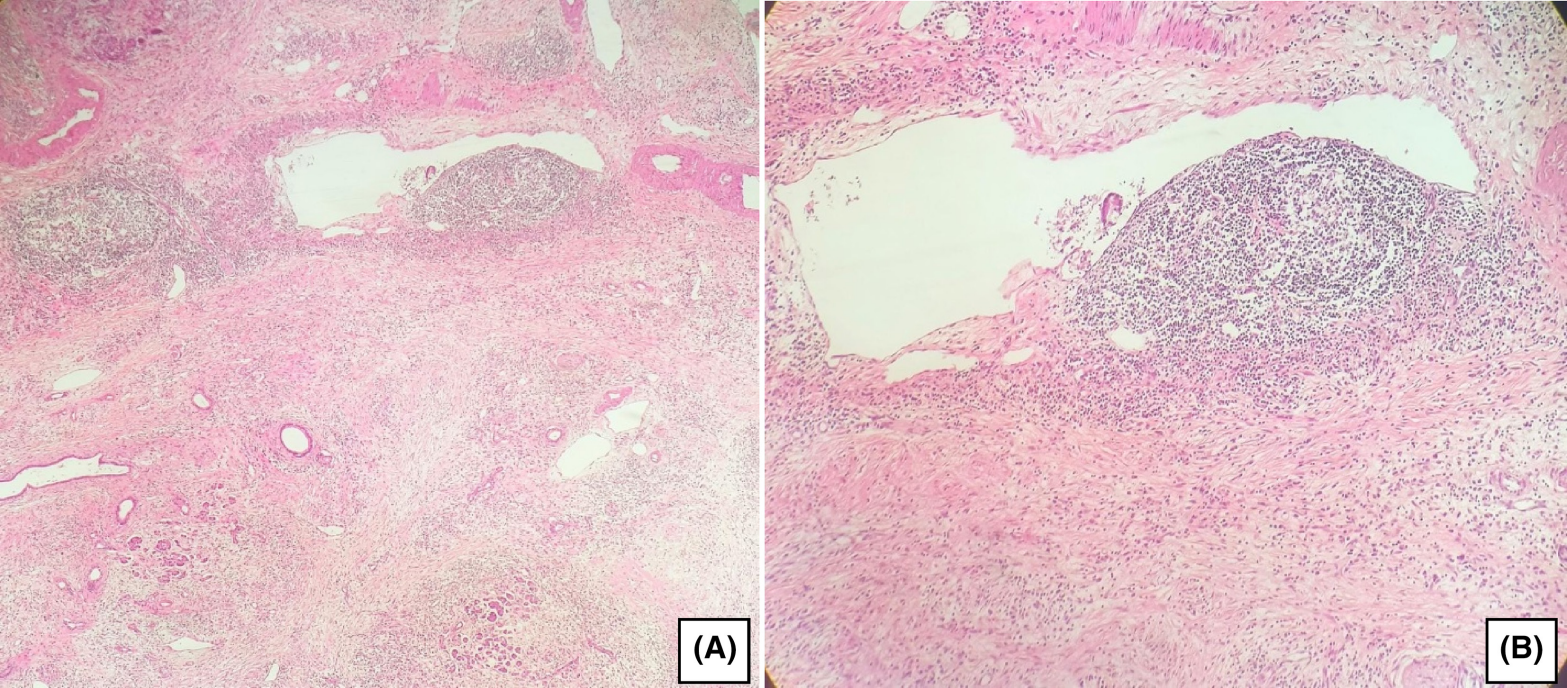
Histopathologic features of autoimmune pancreatitis. This is the same patient as in Figure 3. (A) and (B) Photomicrographs (hematoxylin–eosin [H‐E] stain) from sections of the intrapancreatic ducts show periductal inflammation associated with fibrosis and thickening of the wall. (B) Photomicrograph shows IgG4‐positive plasma cells around the pancreatic duct.

A 35‐year‐old male with no known co‐morbidities, presented with complaints of epigastric pain for 2 days. On examination, the abdomen was mildly tender in the epigastric region otherwise soft and without rebound tenderness. The rest of the examination was unremarkable. Early laboratory investigations including complete blood count, lipase, amylase, and liver function tests were within the normal range (serum amylase was at the upper limit of normal). CT abdomen with contrast showed a mildly enhancing soft tissue density mass involving the head and uncinate process of the pancreas completely encasing the superior mesenteric artery. Mild peripancreatic fat stranding and inflammatory changes extending into the mesentery along with prominent peripancreatic lymph nodes were also noted. The scan was concluded as acute on chronic pancreatitis. The possibility of an intraductal papillary mucinous tumor was also raised. His serum CA19‐9 (non‐detectable to 39 U/mL) and CEA (0–3.0 ng/mL healthy subjects) were 162 and 2.25 respectively. Endoscopic retrograde cholangiopancreatography showed filling defects in the distal common bile duct (CBD) consistent with sludge. CBD was cleared from the sludge with repeat cholangiogram showing no filling defect. The pancreatic duct could not be cannulated. The patient was discharged in a stable condition. CT abdomen performed 4 years later demonstrated the same findings with interval progression. This time the infiltrating lesion was seen encasing the portal vein and hepatic artery (Figure 3). The scan did not show peripancreatic lymphadenopathy or intrahepatic biliary dilatation as would be expected in pancreatic head tumors. Calcifications in the pancreatic parenchyma were suggestive of recurrent attacks of pancreatitis (Figure 3). The conclusion of the scan was a neoplastic lesion with the remote possibility of IgG4‐related disease. CT‐guided transhepatic core biopsy of the pancreatic lesion was performed. Histopathology reported linear cores of fibro‐collagenous tissue exhibiting dense mixed inflammation with small abscesses. Intrapancreatic ducts were seen to show periductal inflammation associated with fibrosis and thickening of its wall. IgG4‐positive plasma cells were seen around the pancreatic duct (Figure 4). Serum IgG4 level was 2960 mg/L supporting IgG4‐related disease. The patient was prescribed steroids after the histopathology proved negative for malignancy. The patient has not paid a follow‐up visit after this.

## 3 DISCUSSION

Pancreatic malignancy is the seventh most important cause of cancer morbidities globally. In 2018, 459,000 new patients were reported with this disease.^11^ In future, it is predicted to outstrip breast cancer in European countries as the third foremost cause of cancer mortality.^12^ Frequent etiological factors include obesity, nicotine exposure and elevated blood glucose levels.^13^, ^14^ It is perturbing for the patients diagnosed with pancreatic mass and they are mostly worried about the type of lesion which makes it essential to counsel appropriately and conduct rapid medical examination to conclude the final diagnosis.

IPT is a title devised by Umiker and Iverson in 1954 due to overlapping symptomatic and radiological findings with pancreatic malignancy.^15^ It is a benign, unusual entity that has a tendency to involve all locations in the body but is most frequently present in orbit and the lung. IPTs may be solitary or multitudinous with a blend of neutrophils and lymphocytes along with an uneven extent of fibrosis, myofibroblastic spindles, necrosis, and formation of granulomas.^16^, ^17^, ^18^

ITP is known to be associated with autoimmune diseases, trauma and fibrosarcoma.^19^, ^20^, ^21^ Autoimmune conditions include IgG4‐associated sclerosing disease where T‐cell and IgG4‐positive plasma cells attack multiple tissues. The manifestations include autoimmune pancreatitis, cholecystitis, tubulointerstitial nephritis, prostatitis, interstitial pneumonia, and sclerosing cholangitis along with lymphadenopathy. Few patients have reported IgG4‐related IPTs in the absence of pancreatitis.^22^, ^23^

IgG4‐related disease presents as a tumor‐like swelling involving the organ it affects. The type 1 form of IgG4‐related disease depicts a type of autoimmune pancreatitis. Patients frequently show up with an acutely developing mass, painless obstructive jaundice, and diffuse organomegaly. Such a presentation can be misunderstood for pancreatic cancer.^24^ IgG4‐related disease has a criteria of a serum IgG4 level >135 mg/dL with around 40% of IgG+ plasma cells being IgG4+ (>10 cells/high‐power field of biopsy sample). The following criterion is valuable, yet not adequately sensitive to diagnose type 1 IgG4‐related autoimmune pancreatitis.^25^

An international agreement was set up by the International Association of Pancreatology regarding the differential diagnosis of the two particular types of autoimmune pancreatitis (types 1 and 2). This can be differentiated based on 5 criteria: (1) imaging changes in the pancreatic parenchyma and duct; (2) serology (for IgG4 and IgG antinuclear antibodies); (3) extrapancreatic involvement; (4) histology; and (5) response to corticosteroid therapy.^26^ Radiological evaluation plays a crucial role in closely studying and identifying these lesions. However, the histopathological review is thought to be a much‐needed step in landing a definitive diagnosis.^27^ CT scans might show a variable appearance of such inflammatory tumors, from hypoattenuated to isoattenuated depending on the muscle and few calcifications might be there in the liver, pancreas, or stomach. MRI images can likely differ, after the introduction of the contrast heterogeneous enhancement. However, this still cannot differentiate these lesions from true pancreatic tumors.^19^

The definitive diagnosis of these lesions depends on the histopathological outcomes; with some specimens being obtained on postsurgical resection. This accounts for around 5%–10% of the pancreatectomies. Steroids are the mainstay of therapy for IgG4‐related pancreatic head pseudotumor. Even in case of recurrence, the standard of care is to administer steroids with the possible addition of other immunosuppressant drugs.^21^, ^27^ Recurrence rate is calculated to be around 18%–40%.^27^ Such recurrent lesions can be associated with local invasion. Resection of such lesions is indicated since they might possess a malignant transformation potential.^21^ Literature suggests few occurrences of spontaneous regression.^21^, ^27^

IPTs are a rarity and a frequent incidental finding during routine radiological examinations. They can also be encountered while investigating for non‐specific clinical features or a detected mass from an unknown source. Definite diagnosis depends on the radiological and histological assessment that can be done postsurgical resection or biopsy. Surgical resection is the first line of treatment if the diagnosis is not clear or was not previously done and is curative in most cases. In the case of autoimmune conditions like IgG4‐related pancreatic disease steroids are the mainstay of therapy.

## 4 CONCLUSION

**FIGURE 5:**
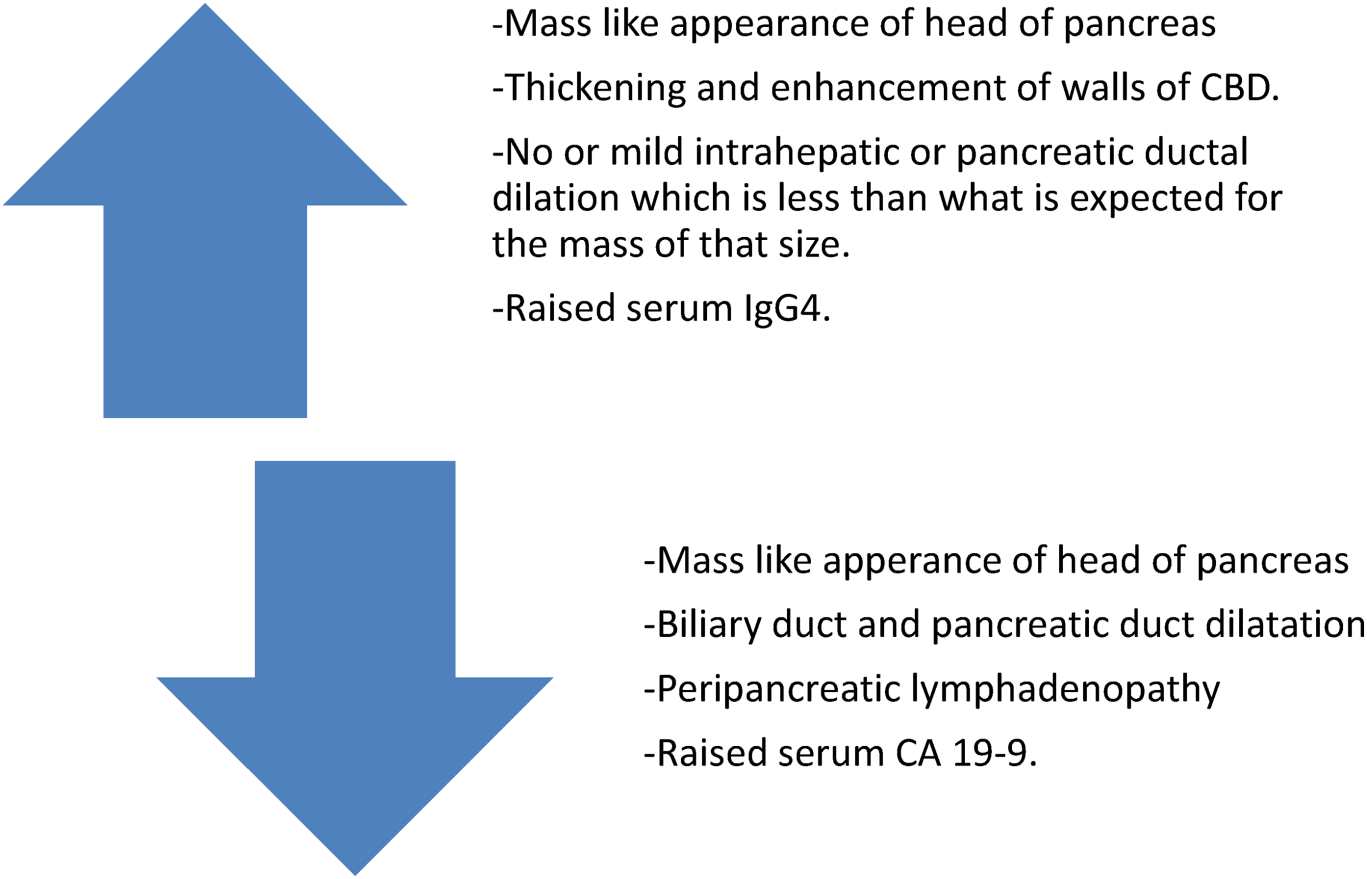
Depicting features favoring pancreatic head pseudotumor (autoimmune/IgG4 related disease) over the pancreatic head tumor.

Pancreatic head involvement in IgG4‐related disease can be mistaken for a pancreatic head tumor. An understanding of the pattern of presentation on cross‐sectional imaging is essential in helping to make the correct diagnosis and direct appropriate management. Imaging findings with raised serum IgG4 levels help in condensing the differentials and avoid any redundant invasive procedure (Figure 5). This can further reduce infections, complications and in turn mortality rate. On the contrary, misdiagnosing this entity can be fatal.

## AUTHOR CONTRIBUTIONS

**Faheemullah Khan:** Conceptualization; investigation; supervision; validation; visualization; writing – original draft; writing – review and editing. **Jehanzeb Shahid:** Conceptualization; validation; writing – original draft; writing – review and editing. **Amna Saleem:** Validation; visualization; writing – original draft. **Uzzam Ahmed Khawaja:** Validation; visualization; writing – original draft. **Wasim Ahmed Memon:** Visualization; writing – original draft. **Uffan Zafar:** Validation; visualization; writing – original draft. **Tariq Abdul Hameed:** Validation; visualization; writing – original draft. **Khabab Abbasher Hussien Mohamed Ahmed:** Validation; visualization; writing – review and editing.

## FUNDING IMFORMATION

Not applicable.

## CONFLICT OF INTEREST STATEMENT

None to declare.

## CONSENT

Written informed consent was obtained from the patients to publish this report in accordance with the journal's patient consent policy.

## Data Availability

The data that support the findings of this study are available from the corresponding author upon reasonable request.
